# Mapping Aptamer Prototypes to the Unmet Gaps of Glioblastoma Care: From Circulating Cells to the Resection Cavity

**DOI:** 10.3390/cancers18213474

**Published:** 2026-10-28

**Authors:** Galina S. Zamay, Natalia A. Luzan, Anastasia A. Koshmanova, Tatiana N. Zamay, Olga S. Kolovskaya, Maxim V. Berezovski, Anna S. Kichkailo

**Affiliations:** 1 Laboratory for Digital Controlled Drugs and Theranostics, Federal Research Center “Krasnoyarsk Science Center of the Siberian Branch of the Russian Academy of Sciences”, Krasnoyarsk 660036, Russia; galina.zamay@gmail.com (G.S.Z.); laskimo@mail.ru (N.A.L.); tzamay@yandex.ru (T.N.Z.);; 2 Laboratory for Biomolecular and Medical Technologies, Prof. V.F. Voino-Yasenetsky Krasnoyarsk State Medical University, Krasnoyarsk 660022, Russia; 3 Department of Chemistry and Biomolecular Sciences, University of Ottawa, Ottawa, ON K1N 6N5, Canada

**Keywords:** glioblastoma, aptamers, residual disease, circulating tumor cells, fluorescence-guided surgery, blood–brain barrier, glioblastoma stem cells, olaptesed pegol

## Abstract

**Background**: Glioblastoma remains poorly controlled despite maximal safe resection, radiotherapy, and temozolomide. Six problems remain unresolved along this pathway: epithelial-marker-based capture can miss glial cells; infiltration extends beyond visible margins; residual disease survives resection; the blood–brain barrier restricts delivery; receptor switching and stem-like states enable escape; and immune control remains inadequate. This review considers aptamer approaches to these limitations while distinguishing experimental candidates from clinical practice. **Methods**: This narrative synthesis incorporates PubMed records, supplementary Google Scholar searches, primary experimental and clinical reports, and registry verification. Evidence is distinguished by tumor type, model, route, and endpoint; no meta-analysis was performed. **Results**: An EpCAM-independent DNA aptamer supports circulating glioma-cell isolation in a small patient series. A near-infrared conjugate labels ex vivo human tissue and animal tumors, but its reported study did not include intraoperative use in patients. Antagonists, photodynamic constructs, and magnetic nanodiscs provide preclinical approaches to tumor-cell control; nanodisc evidence includes separate ascites and glioblastoma studies. Delivery platforms range from receptor-targeted nanoparticles and STAT3 siRNA chimeras to aptamer–drug conjugates selected in orthotopic patient-derived xenografts. Combined receptor blockade and EphA2 targeting address escape experimentally. GLORIA has published phase I/II dose-escalation and bevacizumab-expansion cohorts for the CXCL12-binding L-RNA olaptesed pegol, while a bispecific 4-1BB–osteopontin aptamer provides glioma-specific preclinical immune-directed evidence. **Conclusions**: Selected studies are relevant to each limitation, but heterogeneous evidence and conceptual selection preclude claims of comprehensive coverage or clinical resolution. Local cavity delivery remains a development hypothesis requiring independent safety and efficacy assessment. Aptamers should complement—not replace—established care.

## Simple Summary

Glioblastoma is an aggressive brain cancer that almost always recurs after surgery, radiation, and chemotherapy. Blood tests for other cancers often miss these tumor cells; surgeons cannot always see where the tumor blends into healthy brain tissue; leftover cells remain after an apparently complete operation; and many drugs never reach the brain in useful amounts. This review asks whether short synthetic DNA or RNA molecules called aptamers have already been tested at each of these weak points. Selected studies provide laboratory and early clinical examples relevant to these needs. Some approaches have been tested in glioma models, whereas others have only been demonstrated in other cancers. These findings identify possible research directions but do not show that the clinical needs have been met. The FDA-approved aptamer medicines discussed here are injected into the eye, not into a vein. Applying aptamers locally to the surgical cavity is one development strategy, but its safety and benefit must be established independently. The work is meant to guide researchers toward residual disease rather than toward another list of sequences.

## 1. Introduction

Glioblastoma is an aggressive, highly infiltrative primary brain malignancy in adults [1]. In the CBTRUS report covering diagnoses in the United States in 2018–2022, glioblastoma accounted for 13.7% of all primary brain and other CNS tumors and 52.2% of malignant tumors [2]. The CBTRUS glioblastoma fact sheet published in 2025 reports an average annual age-adjusted incidence of 3.27 per 100,000 population for 2017–2021 [3]. These estimates are population—and classification—dependent and should not be interpreted as a worldwide incidence rate. Recurrence remains a major clinical challenge.

The 2016 WHO classification listed glioblastoma as grade IV and still distinguished IDH-wildtype and IDH-mutant glioblastoma [4]. The 2021 WHO CNS classification restricts the name glioblastoma to IDH-wildtype diffuse astrocytic glioma, WHO grade 4 [5]. IDH-mutant tumors formerly called secondary glioblastoma are now astrocytoma, IDH-mutant, grade 4. We use the current name and keep older epidemiologic series that still used the 2016 label.

Glioblastoma infiltrates surrounding brain, rarely spreads outside the central nervous system, and can disseminate through cerebrospinal fluid. Microscopic disease can remain after apparently complete resection and contribute to recurrence [6,7].

For readers unfamiliar with neuro-oncology, the Stupp regimen is the standard chemoradiotherapy given after maximal safe resection: focal radiotherapy (60 Gy in 30 fractions over six weeks) with daily temozolomide (75 mg/m^2^), followed by six 28-day cycles of adjuvant temozolomide (150–200 mg/m^2^/day on days 1–5) [8]. The pivotal randomized trial showed that this combination improved survival compared with radiotherapy alone [8]. Tumor Treating Fields (TTFields) with maintenance temozolomide are an additional option for eligible patients where available, but were not part of the original regimen [9]. Even so, functional brain boundaries limit surgery, and chemoradiotherapy does not reliably eradicate infiltrative, heterogeneous, and treatment-resistant tumor populations [6,10]. The six limitations discussed below therefore persist along and after this treatment sequence; they are neither stages of disease nor reasons to replace standard care. Imaging and tissue-based molecular assays remain central to diagnosis and treatment planning [11,12].

Contrast-enhanced MRI does not delineate the full extent of glioma infiltration. Complete resection of contrast-enhancing tumor can therefore leave non-enhancing disease, which motivates maximal safety and, in selected patients, supramaximal resection of non-enhancing tumor [7]. Amino-acid PET can supplement MRI in delineating tumor extent [13]. Intraoperatively, 5-aminolevulinic acid and sodium fluorescein can assist tumor visualization, but neither completely delineates microscopic infiltration; their different mechanisms and evidence are discussed below [14,15]. These methods do not eliminate the need to protect functional brain tissue or to develop better approaches to residual disease. Other limitations include lineage-inappropriate circulating-cell capture, heterogeneous drug delivery across the blood–brain barrier, compensatory signaling, stem-like states, and inadequate immune control.

DNA and RNA aptamers selected against glioma cells and glioma-associated receptors offer experimental approaches to limitations that persist despite maximal safe resection and Stupp-based chemoradiotherapy. Recent reviews examine glioma imaging, receptor targeting, therapeutic delivery, and molecular engineering [16,17,18,19,20]. Building on these syntheses, the present review considers selected studies in relation to six persistent limitations of care, distinguishing glioma-specific evidence from findings in other cancers and early clinical testing. This conceptual mapping is not exhaustive and does not imply that any clinical need has been met. Author-group studies are included alongside independent work, not as the sole or most mature evidence. For magnetic nanodiscs, the ascites-tumor study [21] and the separate glioblastoma-specific preclinical report [22] are distinguished; neither establishes clinical efficacy against postoperative residual disease.

This is a narrative review. Unconventional aptamer approaches are presented with their potential advantages and with prospects for clinical use, graded as prototypes rather than as practice.

## 2. Literature Approach

This narrative review examines aptamer approaches in relation to six persistent limitations of glioblastoma care rather than classifying ligands primarily by sequence. Original experimental studies, clinical reports, and reviews were considered, with emphasis on English-language publications. The literature was assembled through searches and revision checks undertaken on 12 September and 19 October 2026. Search concepts combined glioblastoma or glioma with aptamer or SELEX and topic-specific terms: circulating tumor cells, EpCAM, liquid biopsy, fluorescence-guided surgery, magnetic nanodiscs or magnetomechanical treatment, blood–brain barrier, siRNA or chimera, glioma stem cells or EphA2, and CXCL12, olaptesed pegol, or NOX-A12.

PubMed search outputs were consolidated and checked on 19 October 2026. The two exported datasets contained 43 and 2341 records, respectively. Deduplication by PMID identified 34 overlapping records, leaving 2350 unique PubMed entries for relevance assessment. The smaller dataset contained abstracts and comprised 42 records classified as reviews, including book chapters, and one editorial; the larger dataset contained bibliographic citations without abstracts. These counts describe the exported literature pool rather than the number of studies included or the number of abstracts and full texts screened. Recent reviews, including publications from 2025 to 2026, informed the conceptual context and identification of primary reports. Specific claims were checked against primary abstracts and accessible full-text sections; a full-text check is not claimed when only an abstract or bibliographic record was available.

Google Scholar was also searched for additional publications. The interface displayed approximately 1280 results without an article-type restriction and approximately 618 results with the review-article filter applied. These approximate, potentially overlapping estimates were not summed or added to the PubMed count and are not treated as numbers of screened or included publications.

Selection was guided by the mechanistic relevance of an aptamer-based approach to one or more of the six limitations. Reviews provided context, whereas original reports were prioritized for claims about specific constructs, experimental effects, and clinical outcomes. Evidence was distinguished by tumor type, model, route of administration, and endpoint. Glioma-specific findings were separated from results in other cancers; subcutaneous tumors were distinguished from intracranial models; and ex vivo human-tissue observations were distinguished from administration to patients. Author-group studies were considered alongside independent work and were not treated as the sole evidence for an approach.

ClinicalTrials.gov records were reviewed on 19 October 2026 to distinguish aptamer interventions from other investigational glioblastoma treatments. Of the two records supplied for assessment, NCT04677049 was excluded because it evaluates niacin rather than an aptamer [23]. NCT04121455 (GLORIA) was included because it investigates olaptesed pegol in newly diagnosed MGMT-unmethylated glioblastoma [24]. The supplied export listed GLORIA as active, not recruiting. Its published dose-escalation and bevacizumab-expansion cohorts were treated as reports from one registration rather than independent trials [25,26]. Table S1 summarizes the verified registration, published cohorts, other registry-listed strategies, and the excluded record; it is not an exhaustive census of aptamer trials.

Several limitations apply. The available exports do not contain complete search histories, publication-date filters, individual selector roles, or a record-by-record screening log; they therefore do not establish the numbers assessed at abstract and full-text stages. No protocol was registered, no meta-analysis was performed, and exhaustive retrieval or independent duplicate screening is not claimed. Conceptual selection may favor supporting examples and underrepresent negative findings, unsuccessful translation, or competing technologies. Inclusion of the authors’ own studies introduces an additional risk of selection and interpretive bias. The mapping is hypothesis-generating rather than evidence of comprehensive coverage or comparative superiority. Findings in other cancers are not treated as evidence of glioblastoma efficacy, and small uncontrolled clinical cohorts cannot establish comparative benefits or routine adoption.

## 3. The Stupp Pathway and Its Unmet Gaps

Glioblastoma has no validated TNM-style staging system. Spread outside the central nervous system is uncommon, and descriptive clinical “stages” should not be confused with a treatment-determining TNM classification [1,6]. Historically, most tumors labeled glioblastoma presented de novo, usually in older adults [1]. This clinical description is not interchangeable with molecular classification. Under WHO CNS5, glioblastoma is IDH-wildtype, whereas IDH-mutant grade 4 astrocytoma is a distinct entity [5].

The operative unit of care is therefore not a stage but a treatment sequence. The Stupp-based pathway outlined above provides the clinical backbone for this review [8]. Each step leaves an unmet need, hereafter a gap: a limitation that established care does not consistently resolve.

The gaps that structure this review are: (1) circulating detection in the absence of an epithelial lineage marker; (2) intraoperative definition of the infiltrative margin; (3) residual tumor cells after gross-total resection of contrast-enhancing disease; (4) delivery of drugs and therapeutic oligonucleotides across the blood–brain barrier; (5) receptor tyrosine-kinase switching and glioma stem-like compartments; and (6) failure of immune control.

Tissue and imaging remain the diagnostic backbone [11,12]. The persistence of unmet needs despite established care motivates this review, not replacement of the Stupp regimen. Nuzzo et al. provide historical context [27], complemented by recent syntheses of targeting, delivery, diagnostic applications, and molecular engineering [16,17,18,19,20]. Clinical evidence is considered separately using the primary GLORIA reports and registry [24,25,26]. The following sections distinguish experimental feasibility from clinical validation; the existence of a prototype does not establish that the corresponding clinical need has been met.

## 4. Gap 1. Circulating Detection Without an Epithelial Lineage Marker

Tissue-based integrated diagnosis remains the reference standard, while repeated brain sampling is generally unsuitable for longitudinal monitoring. Liquid biopsy—analysis of blood, cerebrospinal fluid, and other fluids for tumor-derived nucleic acids, circulating cells, and extracellular vesicles—is being investigated as a complementary approach [28,29,30,31]. Current reviews emphasize low analyte abundance, matrix-dependent yield, methodological variability, and incomplete standardization; these are distinct from the choice of capture ligand [30,31].

Molecular alterations explored in glioma liquid-biopsy studies include IDH1/IDH2 mutations, MGMT promoter methylation, TERT promoter mutations, and EGFR alterations [32]. Their interpretation depends on tumor class and assay performance: IDH-mutant astrocytoma and IDH-wildtype glioblastoma are distinct categories, and 1p/19q codeletion defines oligodendroglioma rather than glioblastoma [5]. Tissue prevalence should not be equated with detection rates in plasma or cerebrospinal fluid. Cerebrospinal fluid is often more informative for tumor-specific alterations, whereas plasma permits repeat sampling but frequently has lower analyte abundance [30,32].

Glioma-specific studies have demonstrated circulating tumor cells in peripheral blood using hematopoietic-cell depletion, GFAP-based detection with molecular confirmation, and a telomerase-promoter-based assay [33,34,35]. These findings establish hematogenous dissemination, not clinical validation of an aptamer assay. EpCAM-dependent capture was developed primarily for epithelial cancers and is not a reliable lineage-based strategy for glial tumors. Glioma-directed aptamers can instead be selected against tumor-associated binding phenotypes without requiring EpCAM as a predefined marker [36].

**Figure 1:**
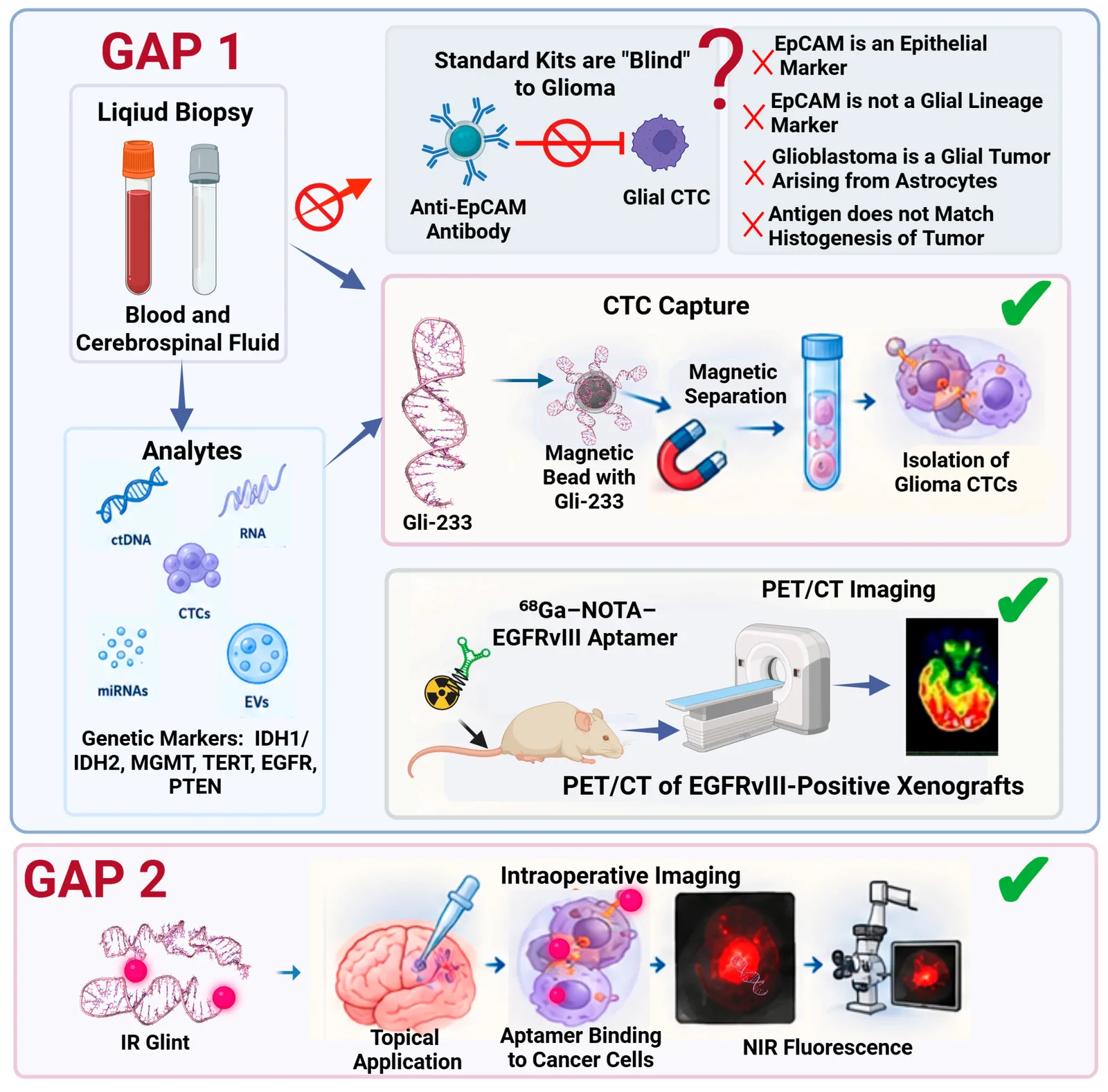
Unmet needs before and during glioblastoma surgery: aptamer prototypes for EpCAM-independent circulating-cell detection (Gap 1) and delineation of the infiltrative margin (Gap 2). The examples do not imply validated clinical benefit.

DNA aptamers Gli-55 and Gli-233 were obtained by cell-SELEX on postoperative human glial tumors with counter-selection against healthy brain and meningioma. They bind glioma cells in vitro, ex vivo, and in vivo. Biotinylated Gli-233 on streptavidin magnetic beads has been used to isolate glioma circulating tumor cells from blood; cerebrospinal fluid is a second matrix [36]. Isolated cells can be enumerated or profiled. Related sequences were subsequently developed into near-infrared probes for preclinical and ex vivo visualization, discussed under Gap 2 [37]. Independent groups have used aptamers as optical or nuclear ligands for glioma cells. A ^68^Ga-labeled NOTA–EGFRvIII aptamer accumulated preferentially in EGFRvIII-positive subcutaneous xenografts; that study reported no specific brain uptake and did not test orthotopic tumors, so it is not evidence of intracranial imaging or liquid-biopsy capture [38]. Aptamer platforms for isolation of circulating tumor cells and extracellular vesicles are reviewed as a general liquid-biopsy technology; glioma-specific analytical validation remains sparse. Single-cell RNA sequencing has shown that primary glioblastoma is transcriptionally heterogeneous [10], which motivates evaluation of marker coverage rather than reliance on one target (Figure 1, Gap 1).

An EpCAM-independent aptamer prototype for circulating glioma cells therefore exists [36]. It demonstrates that a lineage-appropriate ligand can be developed; it does not demonstrate readiness for diagnosis, monitoring, or prognosis in routine care. Such use requires independent analytical validation, prospective clinical validation, and demonstration that the result improves patient management [29,36].

## 5. Gap 2. Molecular Definition of the Infiltrative Margin

Complete resection of contrast-enhancing tumor does not imply clearance of non-enhancing infiltrative disease. In selected patients, supramaximal resection extends beyond the enhancing component, subject to functional boundaries; the RANO resect framework explicitly distinguishes contrast-enhancing and non-enhancing residual tumor volumes [7]. The relevant clinical question for an aptamer probe is therefore its incremental value within contemporary maximal-safe-resection workflows, rather than visibility compared with white light alone (Figure 1, Gap 2).

Existing intraoperative comparators include 5-aminolevulinic acid (5-ALA), which generates intracellular protoporphyrin IX fluorescence, and sodium fluorescein, which primarily reflects blood–brain-barrier disruption [14,15]. Their evidence and regulatory positions are not equivalent: 5-ALA has randomized phase III evidence, whereas fluorescein is used off-label in many settings [14,15]. In a single-center retrospective cohort of 154 patients with IDH-wildtype glioblastoma, Castaldo et al. compared 5-ALA, sodium fluorescein, and their combination. No statistically significant differences were observed in the extent of resection of either contrast-enhancing or FLAIR-hyperintense components. However, 5-ALA was associated with longer progression-free and overall survival than sodium fluorescein, including in adjusted analyses. Because fluorophore allocation depended on institutional availability rather than randomization, these findings should be interpreted as associations rather than proof of a causal survival advantage [39].

Amino-acid PET is an established adjunct for delineating glioma extent beyond contrast enhancement, as addressed by updated RANO/EANO recommendations [13]. Bianconi et al. provided preliminary histopathological and intraoperative fluorescence validation using 25 biopsies from five patients: [18F]DOPA-positive regions beyond enhancement contained substantial neoplastic tissue, with higher tumor-cell density and mitotic activity than FLAIR-only areas [40]. The small, selected cohort does not establish a survival benefit from PET-guided resection. FLAIR abnormality, PET positivity, fluorescence, and microscopic infiltration should not be treated as interchangeable boundaries.

One documented implementation is topical. IR-Glint is a mixture of the Cy7.5-labeled aptamers Glint2-1 and Glint5-1, redesigned from Gli-233 and Gli-55. Near-infrared emission can be detected with an appropriate surgical fluorescence microscope module. In the mouse experiment, topically applied IR-Glint penetrated approximately 3–4 mm into brain tissue within 3 min; this does not establish staining depth or margin accuracy in human brain [37]. Topical application to exposed brain was investigated in animal experiments; its clinical exposure profile and safe repeat application remain to be established. The reported study demonstrated binding to patient-derived glioma cultures and ex vivo human glioma tissue, and visualization of human-derived glioma in mouse and rabbit models. It did not demonstrate intraoperative administration to patients. The proposed advantage is ligand-mediated tumor-associated recognition, not demonstrated superiority over 5-ALA or sodium fluorescein. The cited study does not establish improved extent of resection or survival compared with either clinical fluorophore. Validation would require spatially registered, blinded histopathology; sensitivity and specificity in enhancing and non-enhancing tissue; incremental detection beyond existing fluorescence; and neurological safety [14,15,37,39,40].

Systemically administered aptamer imaging constructs illustrate the same ligand logic without establishing intraoperative clinical use. The ^68^Ga–NOTA–EGFRvIII aptamer reports EGFRvIII-positive subcutaneous xenografts on PET and is not evidence of orthotopic brain imaging [38]. Gol1, a computationally redesigned derivative of U2, was evaluated in patient-derived glioblastoma cultures by proliferation and transcriptomic assays; it is not an intraoperative stain or a clinically validated AI-designed ligand [41].

In the operative field, an aptamer remains vulnerable to low epitope density, nuclease-mediated degradation, irrigation washout, and off-target binding. Those failure rates have not been quantified in a controlled human margin study [37]. A topical aptamer–near-infrared conjugate is therefore a prototype for molecular margin definition. The cited visualization study provides preclinical and ex vivo feasibility evidence, not validation as a clinical margin standard [37].

## 6. Gap 3. Residual Cells After Gross-Total Resection

Residual infiltrative cells may persist after complete removal of the enhancing tumor and even after supramaximal resection, because functional brain boundaries limit tissue removal [7]. The approaches discussed below are candidate adjuncts to maximal safe surgery, not substitutes for further safely achievable resection or established adjuvant treatment.

Some aptamers are themselves functional antagonists. AS1411, which binds nucleolin, a protein enriched on rapidly dividing cells including glioblastoma, can arrest the cell cycle, inhibit DNA synthesis, and shift apoptotic regulators; Cheng et al. reported G2/M arrest and apoptosis in glioma cells and reduced growth with prolonged survival in mice bearing subcutaneous U87 xenografts. This is ectopic preclinical evidence, not intracranial residual-cavity efficacy. A separate phase II study in metastatic renal-cell carcinoma is clinical evidence from a different cancer [42,43]. CL4, directed against EGFR including EGFRvIII, inhibits receptor signaling, migration, invasion, and proliferation; under the conditions tested, CL4 alone or with Gint4.T did not induce detectable apoptosis [44]. Gint4.T, directed against PDGFRβ, can arrest the cell cycle and promote differentiation in experimental systems; its reported in vivo tumors were subcutaneous [45]. These ligands illustrate inhibition of tumor-cell signaling or growth; they do not by themselves establish cavity-directed physical clearance.

Magnetomechanical disruption uses anisotropic magnetic discs to convert an alternating or rotating magnetic field into mechanical forces on associated cells, without requiring a cytotoxic payload or magnetic heating [46]. The ascites study used aptamer-functionalized discs with intraperitoneal administration in an Ehrlich ascites carcinoma model [21]. Targeting enhanced the effect, but nonspecific oligonucleotide-coated discs also caused some damage. This non-glioma experiment does not establish absolute selectivity, intracranial distribution, or neurological safety. A separate glioblastoma-directed report must be considered independently, rather than inferred from the ascites results.

Fedotovskaya et al. reported glioblastoma-directed preclinical work using aptamer-functionalized magnetic nanodiscs activated by a non-heating alternating magnetic field [22]. This provides disease-specific evidence beyond the ascites model, but does not establish clinical efficacy, postoperative cavity distribution, or neurological safety. Translation would require model-specific confirmation, characterization of off-target mechanical effects, field parameters, tissue retention, and reproducible manufacture.

**Figure 2:**
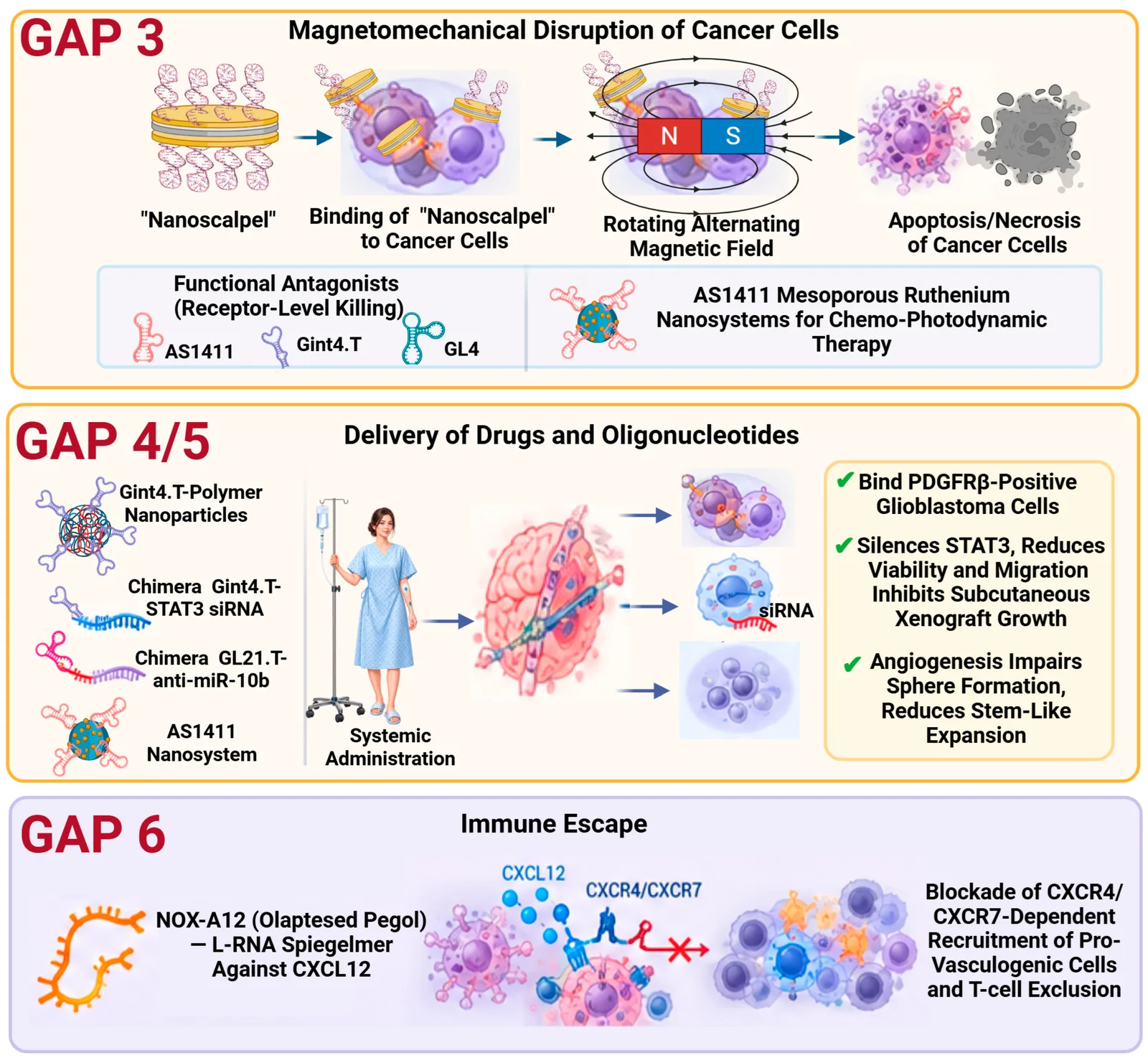
Unmet needs in glioblastoma care after surgery: aptamer prototypes for residual-tumor control (Gap 3), delivery and receptor/stem-like escape (Gaps 4/5), and immune or microenvironmental modulation (Gap 6). Evidence levels vary between approaches.

Aptamer-targeted photodynamic or chemo-photodynamic constructs provide a third prototype, in which the oligonucleotide directs a photosensitizer or a combined chemo-photodynamic payload to glioma cells (Figure 2, Gap 3). Transferrin and AS1411 dual-modified mesoporous ruthenium nanosystems have been explored for redox-controlled, targeted chemo-photodynamic therapy of glioma in preclinical work [47]. These still remain laboratory systems.

Antagonistic, magnetomechanical, and photodynamic constructs may therefore be investigated as adjuncts to glioma surgery, but the cited studies do not establish a clinically validated cavity treatment [21,22,42,44,45,47]. Non-glioma proof of concept, glioma-specific preclinical activity, and postoperative clinical efficacy remain separate evidence categories.

## 7. Gap 4. Delivery of Drugs and Therapeutic Oligonucleotides Across the Blood–Brain Barrier

Drug delivery is one of several major constraints in glioblastoma treatment, alongside biological resistance and heterogeneity [6,48,49]. Aptamers can act as targeting ligands on drugs or nanoparticles, or as internalizing components of therapeutic-oligonucleotide chimeras. Recent reviews synthesize both formats [17,19,50], but the delivery and functional performance of an individual construct must be assessed from its primary report.

Polymer nanoparticles conjugated to Gint4.T, a nuclease-resistant RNA aptamer against PDGFRβ, have been shown to bind PDGFRβ-positive glioblastoma cells, to cross an in vitro blood–brain barrier model, and to accumulate in orthotopic U87MG tumors after systemic administration [51]. Gint4.T itself antagonizes PDGFRβ [45]; on a nanoparticle it additionally serves as a delivery address. The same aptamer has been fused to a STAT3 siRNA. The resulting chimera, Gint4.T-STAT3, delivers the siRNA to PDGFRβ-positive glioblastoma cells, silences STAT3, reduces viability and migration in vitro, and inhibits subcutaneous xenograft growth and angiogenesis [52]. In patient-derived glioblastoma stem-like cells the chimera impairs sphere formation; combination with an AXL-targeted anti-miR-10b construct (GL21.T–anti-miR-10b) further reduces stem-like expansion [53]. These studies demonstrate receptor-gated oligonucleotide delivery. They do not demonstrate that an intravenous chimera reaches infiltrative human glioblastoma at a therapeutic index acceptable for registration.

More recent work selected DNA aptamers in an orthotopic patient-derived xenograft model, with preferential tumor accumulation detected 4 h after intraperitoneal administration [54]. A subsequent study incorporated an MMAE-linked cargo into the selection library and characterized aptamer–drug conjugate binding, biodistribution, and pharmacokinetics [55]. Importantly, the latter report found that adding a linker–cargo to previously selected aptamers could disrupt cell binding, illustrating why the final conjugate must be validated rather than assumed to retain the properties of its parent ligand. These selection and homing results are not evidence of clinical efficacy.

AS1411 has also been used as a targeting component of drug-loaded or multifunctional systems, including the dual-targeted ruthenium platform described under Gap 3 [47]. Aptamer recognition, internalization, cargo release, and pharmacological action are distinct steps; successful binding does not establish delivery of an active payload to infiltrative tumor cells. This distinction applies to both nanoparticle systems and therapeutic-oligonucleotide chimeras [17,19,52].

Limits are structural rather than conceptual. Nuclease susceptibility and clearance depend on sequence chemistry, size, and formulation. PEGylation can reduce renal filtration, and L-RNA Spiegelmers resist natural nucleases; these properties must be measured for each construct [56]. Chemical modifications and nanocarriers also introduce formulation, toxicity, and manufacturing constraints. Heterogeneity of PDGFRβ, nucleolin, or EGFR/EGFRvIII means that a single aptamer will not address every clone. Blood–brain barrier crossing shown in a transwell assay or in a highly leaky xenograft does not equal delivery throughout the human infiltrative margin. An aptamer–drug or aptamer–siRNA prototype for Gap 4 therefore exists (Figure 2, Gap 4/5) [51,52]. These preclinical reports do not establish clinical efficacy or routine use of such conjugates in glioblastoma [51,52,53].

## 8. Gap 5. Receptor Switching and Glioma Stem-like Compartments

A single surface biomarker does not represent glioblastoma. Single-cell RNA sequencing of primary tumors has shown coexisting transcriptional states within one mass. This heterogeneity motivates evaluation of target coverage, but it does not by itself establish escape from any particular ligand [10]. Two interlocking biological problems occupy this gap: compensatory receptor tyrosine-kinase signaling after blockade of EGFR or EGFRvIII, and a stem-like subset that reconstitutes the tumor after cytoreduction (Figure 2, Gap 4/5). Aptamer prototypes exist for both. Neither is a clinical standard.

CL4 is a neutralizing RNA aptamer against EGFR, including the constitutively active EGFRvIII mutant. In glioblastoma cells it reduces EGFRvIII autophosphorylation and limits migration, invasion, and proliferation [44]. Gint4.T is a neutralizing RNA aptamer against PDGFRβ; it inhibits receptor signaling and glioblastoma-derived tumor growth in experimental systems [45]. Gint4.T inhibits PDGF-BB-induced transactivation of wild-type EGFR, and combined CL4/Gint4.T treatment enhanced receptor inhibition and reduced subcutaneous U87MG-derived xenograft growth [45]. In a separate study, CL4 increased PDGFRβ expression in EGFRvIII-expressing cells, and the combination more strongly inhibited proliferation than either aptamer alone; the EGFRvIII-specific cellular experiment is distinct from the 2014 xenograft study [44]. That combination is the aptamer counterpart of a documented escape route: EGFR blockade bypassed through PDGFRβ. It remains a cell- and xenograft-level prototype.

Glioma stem-like cells (GSCs) are a second, overlapping problem. The 2′-fluoropyrimidine RNA aptamers 40L and the truncated 30-mer A40s bind EphA2, discriminate GSCs from more differentiated glioma cells, and inhibit GSC growth, stemness, and migration [57]. A40s–IR700DX retained binding and induced light-dependent cell death in vitro. A40s–ICG was assessed after intravenous administration in three mice bearing subcutaneous GL261-luc2 stem-like tumors (one free-ICG control and two conjugate-treated animals); this does not establish intracranial delivery or in vivo photodynamic efficacy [58]. The Gint4.T–STAT3 chimera discussed under Gap 4 likewise impairs sphere formation in patient-derived GSCs [52,53]. These data place an EphA2 aptamer and a PDGFRβ-gated STAT3 silencer on the stem-like compartment. They do not place either ligand in guideline care.

Structural optimization is a separate evidence layer. Diakogiannaki et al. combined computational and experimental methods to investigate the A40s fold and its interaction with EphA2 [59]. This informs variant design but does not establish improved clinical efficacy. Similarly, GR20 retained cell binding and uptake after truncation of the anti-EGFR aptamer U31, yet showed weaker antiproliferative effects in the tested glioblastoma cultures [60]. Binding, uptake, and growth inhibition should therefore be evaluated as distinct endpoints.

Heterogeneity cuts both ways. Transcriptional heterogeneity motivates checking target coverage across patients and cell states, rather than assuming that a single receptor represents the entire tumor [10]. Postoperative-tissue SELEX, as used for Gli-family DNA aptamers [36], samples a complex tumor-associated phenotype; superiority over purified-target selection requires a direct comparison, and it does not remove the need for multi-patient validation, pharmacokinetics, or safety. An aptamer prototype for receptor switching (CL4 plus Gint4.T) and an aptamer prototype for GSCs (A40s/EphA2) therefore exist [44,45,57]. The supporting studies remain preclinical and do not establish a validated dual-receptor or GSC-directed aptamer regimen for patients [44,45,57,58].

## 9. Gap 6. Failure of Immune Control

Glioblastoma has a strongly immunosuppressive microenvironment, with important myeloid-cell contributions and heterogeneous immune-cell function [6,61]. Non-aptamer approaches have also been investigated: a phase II study combined autologous tumor-lysate-pulsed dendritic-cell vaccination with fluorescence-guided surgery and radiochemotherapy, but its uncontrolled design did not establish a comparative survival benefit [62]. For aptamers, the evidence considered here includes CXCL12-directed microenvironmental intervention and a bispecific immunostimulatory construct. These mechanisms should not be conflated, and neither constitutes an established glioblastoma standard.

Olaptesed pegol (NOX-A12) is a CXCL12-binding L-RNA Spiegelmer investigated for disruption of post-radiotherapy revascularization and microenvironmental signaling. In GLORIA (NCT04121455), the published dose-escalation cohort comprised ten patients with newly diagnosed, incompletely resected or biopsied, MGMT-unmethylated glioblastoma receiving radiotherapy and continuous intravenous NOX-A12 at 200, 400, or 600 mg/week. No dose-limiting toxicities or treatment-related deaths were observed, and 600 mg/week was selected as the recommended phase II dose [25]. The bevacizumab expansion has since been published as a full peer-reviewed report: six additional patients received radiotherapy, NOX-A12 at 600 mg/week, and bevacizumab [26]. Median progression-free and overall survival from treatment initiation were 9.1 and 19.9 months, respectively, compared with 5.7 and 12.7 months in the earlier dose-escalation cohort. These exploratory, nonrandomized comparisons are vulnerable to small-sample uncertainty and differences between sequential cohorts; they do not establish comparative efficacy, immune restoration, or standard-of-care status [26]. The registry also describes other expansion strategies, including pembrolizumab; these should not be conflated with the published bevacizumab cohort [24].

A glioma-specific immunostimulatory prototype is the 4-1BB–osteopontin bispecific aptamer, tested intravenously in mice bearing established intracerebral GL261 tumors, where median survival increased by 68% [63]. This is preclinical immune-directed evidence, distinct from the CXCL12/vascular mechanism investigated in GLORIA. Gint4.T–STAT3 and GL21.T–anti-miR-10b were evaluated for tumor-cell or GSC targeting, STAT3 silencing, and growth or sphere inhibition; those studies do not demonstrate delivery to immune cells or restoration of antitumor immunity [52,53].

Gap 6 therefore includes both early human evidence from GLORIA and a glioma-specific preclinical immunostimulatory aptamer (Figure 2, Gap 6) [25,26,63]. GLORIA provides safety and exploratory efficacy signals, not randomized evidence of clinical benefit or proof that immune failure has been resolved [25,26]. Table 1 summarizes the unmet gaps in the Stupp pathway, the corresponding aptamer prototypes and their clinical status.

## 10. Outlook: Local Versus Systemic Translational Paths

Nuclease susceptibility and clearance depend on sequence chemistry, size, and formulation. PEGylation can reduce renal filtration, whereas L-RNA Spiegelmers resist natural nucleases [56]. Two clinically developed aptamer medicines illustrate how compartmental delivery has been handled in ophthalmology. Pegaptanib sodium, a PEGylated RNA aptamer against VEGF165, was approved by the FDA in 2004 for neovascular age-related macular degeneration and is given by intravitreal injection [56,64]. Avacincaptad pegol, a PEGylated RNA aptamer against complement C5, was approved in 2023 for geographic atrophy secondary to age-related macular degeneration, again by the intravitreal route; the pivotal GATHER2 trial used monthly 2 mg injections [65,66]. Neither product is an oncology indication. Their record shows that an aptamer can become a registered drug when delivered into a confined anatomical compartment at a low local dose, but does not establish safety or efficacy for delivery into a different anatomical compartment.

By contrast, a few systemically administered cancer aptamers have been registered in clinical trials. The nucleolin-targeting DNA aptamer AS1411 entered Phase II studies in metastatic renal-cell carcinoma with acceptable safety and limited activity in an unselected population (one objective response among 35 patients) [43]. Intravenous exposure is the harder development problem. Olaptesed pegol provides the directly documented glioblastoma interventional example in this review. GLORIA uses continuous intravenous treatment with radiotherapy, with or without additional agents, and has published phase I/II dose-escalation and bevacizumab-expansion cohorts [24,25,26]. Its development should be distinguished from regulatory approval or routine adoption. The limited activity of AS1411 in renal-cell carcinoma does not by itself establish a common pharmacokinetic cause of failure for all systemic aptamers.

Glioblastoma surgery exposes the resection cavity and adjacent tissue, providing a potential route for topical or intracavitary agents without initial systemic blood–brain-barrier passage. This is a development rationale, not evidence of faster clinical translation or adequate penetration throughout the infiltrative margin. Near-infrared aptamer evidence remains preclinical and ex vivo [37]. Magnetic-nanodisc studies now include both an ascites model and a separate glioblastoma-directed report [21,22], but postoperative cavity efficacy and safety remain unestablished. Ex vivo circulating-cell assays do not require intravenous aptamer administration and should not be grouped with systemic delivery platforms.

This is a pharmacokinetic argument, not a claim of clinical efficacy. The brain is not the vitreous: cerebrospinal-fluid washout, nucleases in the operative field, edema, eloquent cortex, and combination-product rules for a dye or a magnetic disc remain. Local use does not remove the need for GMP synthesis, impurity control, local neurotoxicity studies, or a controlled comparison with existing intraoperative fluorophores. Local and systemic formats should be evaluated on their own delivery, target-engagement, safety, manufacturing, and patient-benefit requirements; the present review does not establish that one route will reach clinical adoption sooner [21,37,56].

## 11. AI-Assisted Target Prioritization and Aptamer Optimization

Recent reviews discuss chemical stabilization, multivalent formats, and computational design [16,20]. Artificial intelligence (AI) and data-driven modeling could support this gap-oriented framework by linking patterns of treatment persistence to target selection and aptamer optimization. Longitudinal single-cell and spatial multi-omics have identified recurrent glioblastoma cell states and tumor–microenvironment interactions under therapy; machine-learning analyses of such datasets could help prioritize cellular compartments and escape pathways relevant to residual disease [67]. Candidate cell-surface antigens could then be ranked using tumor-versus-normal expression, coverage across malignant cell states, persistence at recurrence, and localization at the infiltrative margin; however, transcript abundance alone does not establish accessible surface protein or suitability for aptamer targeting [10,67]. At the ligand-design stage, experimentally demonstrated approaches include RaptGen, which generates and truncates RNA aptamer candidates from high-throughput SELEX data and supports activity-guided optimization, and interpretable generative models trained on SELEX sequence ensembles [68,69]. Secondary-structure prediction, docking, and molecular-dynamics simulations may help prioritize variants for testing. Gol1 and structural investigation of A40s provide glioblastoma-relevant examples of computationally informed design, not clinical validation of AI-generated ligands [41,59]. An iterative design–test–learn strategy could connect candidate generation with measured binding, selectivity, and functional activity. These applications remain prospective: the cited machine-learning studies do not establish clinical efficacy of AI-designed glioblastoma aptamers, and computational scores should not be equated with target engagement or clinical benefit.

## 12. Conclusions

This narrative review maps selected aptamer prototypes and mechanistically relevant proof-of-concept studies to six unmet needs in glioblastoma care [25,26,36,37,45,51,57]. The mapping is hypothesis-generating, not an exhaustive demonstration of coverage, clinical equivalence, or superiority over other ligand classes. Evidence derived outside glioma is explicitly distinguished from glioma-specific evidence.

Current comparators include fluorescence-guided surgery with 5-ALA or sodium fluorescein, maximal safe or selected supramaximal resection, and amino-acid PET-supported delineation of non-enhancing tumor [7,13,14,15,39,40]. Aptamer-based probes must demonstrate added value against these workflows. Most therapeutic constructs discussed here remain preclinical or extra-glioma. For olaptesed pegol, both GLORIA dose escalation and the bevacizumab expansion have full clinical reports, but the small nonrandomized cohorts do not establish a glioblastoma standard of care [25,26].

Intravitreal aptamer medicines show that compartmental delivery can be clinically feasible [64,65], but this does not establish the safety, distribution, or efficacy of delivery into a brain resection cavity. Local margin and cavity formats warrant investigation alongside systemic approaches, with independent validation of author-group examples and explicit testing against current clinical comparators.

Translation requires standardized assays and manufacturing, transparent grading of cell-, animal-, and human-level evidence, prospective safety assessment, and appropriately controlled clinical studies. Aptamers should be developed as potential complements to maximal safe surgery, chemoradiation, and other indicated care rather than as replacements. The proposed map remains a research agenda, not evidence that the identified gaps have been clinically closed.

## Figures and Tables

**Table 1:** Unmet gaps in the Stupp pathway, aptamer prototypes, and clinical status. Assignment of a prototype to a gap is not equivalent to clinical closure.

Gap	Aptamer Prototype(s)	Key Evidence	Clinical Status
1. Circulating detection without an epithelial lineage marker	Gli-233 magnetic CTC capture; Gli-55 staining	Circulating glioma-cell isolation in a small mixed-grade astrocytoma series [36]. No demonstrated clinical utility from this series.	Research prototype; analytical and clinical validation still required.
2. Intraoperative definition of the infiltrative margin	IR-Glint (Glint2-1/Glint5-1–Cy7.5); Gol1 (not an intraoperative stain)	Ex vivo human tissue and human-derived glioma in mouse/rabbit models [37]; Gol1 cell assays [41]. No demonstrated superiority to 5-ALA or fluorescein [14,15,39].	Preclinical and ex vivo feasibility; not demonstrated intraoperative use in patients
3. Residual cells after gross-total resection	AS1411; CL4; Gint4.T; aptamer-coated magnetic nanodiscs; AS1411 chemo-photodynamic nanosystems	AS1411: glioma cells and subcutaneous U87 xenografts [42]. CL4/Gint4.T: receptor and growth effects, not uniform apoptosis [44,45]. Magnetic discs: Ehrlich ascites carcinoma [21] and separate glioblastoma-directed preclinical work [22]. Chemo-photodynamic platform: glioma models [47].	Preclinical glioma and non-glioma evidence. AS1411 phase II in renal-cell carcinoma [43]. Nanodiscs are not clinically validated for postoperative glioblastoma residual disease.
4. Delivery across the BBB	Gint4.T nanoparticles; Gint4.T–STAT3; GL21.T–anti-miR-10b; in vivo-selected DNA aptamers and MMAE-conjugated libraries	Receptor-targeted nanoparticles and oligonucleotide chimeras [51,52,53]; orthotopic PDX selection and homing [54,55].	Preclinical delivery/selection evidence; no clinical benefit established by these reports.
5. Receptor switching and GSCs	CL4 + Gint4.T; A40s/EphA2; A40s–IR700DX (in vitro PDT); A40s–ICG (exploratory subcutaneous imaging)	CL4/Gint4.T signaling and growth studies [44,45]; A40s/EphA2 function and structure [57,59]; dye-specific experiments [58].	Preclinical only; subcutaneous imaging is not intracranial efficacy
6. Failure of immune control	Olaptesed pegol (NOX-A12) + radiotherapy ± bevacizumab (GLORIA); 4-1BB–OPN bispecific aptamer	GLORIA dose escalation (n = 10) [25] and bevacizumab expansion (n = 6) [26]; one registration [24]. 4-1BB–OPN: intracerebral GL261 mice, median survival +68% [63].	Early phase I/II clinical investigation plus a separate preclinical immune prototype. The registry export lists GLORIA as active, not recruiting; not established standard care.

Abbreviations: 5-ALA, 5-aminolevulinic acid; ICG, indocyanine green; BBB, blood–brain barrier; CTC, circulating tumour cell; EphA2, ephrin type-A receptor 2; GSC, glioma stem cell; MMAE, monomethyl auristatin E; PDT, photodynamic therapy; PDX, patient-derived xenograft; STAT3, signal transducer and activator of transcription 3; miR-10b, microRNA-10b; NOX-A12, olaptesed pegol; GLORIA, the phase I/II trial acronym of NOX-A12 in glioblastoma. Stupp pathway: standard post-surgical radiotherapy with concomitant and adjuvant temozolomide for newly diagnosed glioblastoma.

## Data Availability

No new experimental data were generated. Supplementary Table S1 summarizes the registry records and published GLORIA cohorts assessed for this review. The PubMed exports supplied for the update contained 43 and 2341 records, with 34 shared PMIDs and 2350 unique entries after consolidation.

## Supplementary Materials

The following supporting information can be downloaded at https://www.mdpi.com/article/10.3390/cancers18213474/s1, Table S1: Registry evidence assessed on 19 October 2026 and linked clinical publications.

## Abbreviations

The following abbreviations are used in this manuscript:

IDH	Isocitrate dehydrogenase
MRI	Magnetic resonance imaging
CT	Computed tomography
PET	Positron emission tomography
SELEX	Systematic evolution of ligands by exponential enrichment
EpCAM	Epithelial cell adhesion molecule
EphA2	Ephrin type-A receptor 2
CXCL12	C-X-C motif chemokine ligand 12
MGMT	O6-methylguanine-DNA methyltransferase
TERT	Telomerase reverse transcriptase
EGFR	Epidermal growth factor receptor
PTEN	Phosphatase and tensin homolog
PDGFRβ	Platelet-derived growth factor receptor beta
STAT3	Signal transducer and activator of transcription 3
AXL	AXL receptor tyrosine kinase
GSCs	Glioma stem-like cells
CXCR4	C-X-C motif chemokine receptor 4
CXCR7	C-X-C motif chemokine receptor 7
AI	Artificial intelligence
TTFields	Tumor Treating Fields
5-ALA	5-Aminolevulinic acid
OPN	Osteopontin
GBM	Glioblastoma
BBB	Blood–brain barrier
PDX	Patient-derived xenograft
MMAE	Monomethyl auristatin E
PDT	Photodynamic therapy
