# Supplementary material for: Mapping Aptamer Prototypes to the Unmet Gaps of Glioblastoma Care: From Circulating Cells to the Resection Cavity
Source: Cancers (Basel). 2026 Oct 28;18(21):3474. doi: 10.3390/cancers18213474 (PMC13761710; doi:10.3390/cancers18213474)
Supplement: Supplementary file 1 [file cancers-18-03474-s001.zip › cancers-4640894-supplementary.pdf]

cancers

MDPI

Supplementary Materials

Table S1. Registry evidence assessed on 19 October 2026 and linked clinical publications.

Registration / Reference	Population and intervention	Evidence / cohort size	Disposition and clinical status
NCT04121455 (GLORIA): registry [24]	Newly diagnosed MGMT-unmethylated glioblastoma. Eligibility varies by arm; registry lists olaptesed pegol, radiotherapy, bevacizumab, pembrolizumab, and temozolomide.	One registration with dose-escalation, expansion, and further combination/comparator strategies.	Included aptamer registration. Active, not recruiting in the supplied export. Not all listed interventions are administered together.
GLORIA: dose-escalation publication [25]	Radiotherapy + continuous IV NOX-A12 at 200, 400, or 600 mg/week; incompletely resected or biopsy-only cohort.	n = 10. No dose-limiting toxicity or treatment-related deaths; recommended phase II dose 600 mg/week.	Published cohort within NCT04121455, not a separate trial. Does not establish completion of the entire registration.
GLORIA: bevacizumab expansion [26]	Radiotherapy + NOX-A12 at 600 mg/week + bevacizumab.	n = 6. Median PFS 9.1 months; OS 19.9 months from treatment initiation.	Published, nonrandomized expansion. Exploratory comparisons; not evidence of clinical standard or immune restoration.
GLORIA: other registry-listed strategies [24]	Includes pembrolizumab and later dose-development/combination/comparator arms.	Protocol listing is not an outcome report. No efficacy result is inferred here for an unreported arm.	Not counted as additional registrations; do not equate with the published bevacizumab cohort.
NCT04677049: Study of Niacin in Glioblastoma [23]	Niacin CRT added to first-line radiotherapy and temozolomide in newly diagnosed IDH-wildtype glioblastoma.	The supplied export lists an interventional study; this is not an aptamer intervention.	Excluded from the aptamer-trial set. Active, not recruiting in the supplied export.

 https://doi.org/10.3390/cancers18213474
